# Endothelial STAT3 Modulates Protective Mechanisms in a Mouse Ischemia-Reperfusion Model of Acute Kidney Injury

**DOI:** 10.1155/2017/4609502

**Published:** 2017-10-17

**Authors:** Shataakshi Dube, Tejasvi Matam, Jessica Yen, Henry E. Mang, Pierre C. Dagher, Takashi Hato, Timothy A. Sutton

**Affiliations:** Department of Medicine, Division of Nephrology, Indiana University School of Medicine, Indianapolis, IN, USA

## Abstract

STAT3 is a transcriptional regulator that plays an important role in coordinating inflammation and immunity. In addition, there is a growing appreciation of the role STAT3 signaling plays in response to organ injury following diverse insults. Acute kidney injury (AKI) from ischemia-reperfusion injury is a common clinical entity with devastating consequences, and the recognition that endothelial alterations contribute to kidney dysfunction in this setting is of growing interest. Consequently, we used a mouse with a genetic deletion of *Stat3* restricted to the endothelium to examine the role of STAT3 signaling in the pathophysiology of ischemic AKI. In a mouse model of ischemic AKI, the loss of endothelial STAT3 signaling significantly exacerbated kidney dysfunction, morphologic injury, and proximal tubular oxidative stress. The increased severity of ischemic AKI was associated with more robust endothelial-leukocyte adhesion and increased tissue accumulation of F4/80^+^ macrophages. Moreover, important proximal tubular adaptive mechanisms to injury were diminished in association with decreased tissue mRNA levels of the epithelial cell survival cytokine IL-22. In aggregate, these findings suggest that the endothelial STAT3 signaling plays an important role in limiting kidney dysfunction in ischemic AKI and that selective pharmacologic activation of endothelial STAT3 signaling could serve as a potential therapeutic target.

## 1. Introduction

Acute kidney injury (AKI) is a costly clinical entity associated with significant morbidity and mortality [1–3]. Ischemia-reperfusion injury (IRI) is one of the most common contributors to the development of AKI in a variety of clinical scenarios and is a primary factor promoting the onset of delayed graft function following transplantation [4–6]. The pathophysiology of ischemic AKI involves a complex interplay between epithelial tubular injury, inflammation, and microvascular dysfunction that culminates in a decrement of glomerular filtration rate (GFR) that is the ultimate common denominator of AKI [7]. Acute alterations in the kidney microvascular endothelium have been observed in animal models of AKI [8–11], and these alterations confer a variety of functional consequences that can contribute to diminished GFR. The endothelial cells of the peritubular microcirculation play a key role in the regulation of many important vascular functions including blood flow rates, blood coagulation and fibrinolysis, leukocyte attachment, and the transcapillary passage of cells and different molecules from the blood into the interstitium. Endothelial cells are located in close proximity to other cell types in the kidney and thus are keenly positioned to be in constant dialog not only with circulating cells in the blood but with adjacent smooth muscle cells, pericytes, tissue-resident leukocytes, and tubular epithelial cells. Furthermore, there is an emerging appreciation that cross-talk signaling to the endothelium is necessary for the maintenance of microvascular structure in the kidney [12–14]. However, little is known about the mechanisms that lead to alterations of microvascular endothelial function in the setting of ischemic AKI.

The signal transducer and activator of transcription (STAT) family of proteins is the principal signal transducers for many cytokines and growth factors in mammalian cells, and it plays an important regulatory role in the cellular response to acute stress such as occurs in IRI [15]. The STAT family of proteins is comprised of at least seven different members in mammals, and as the name implies, they are important for signal transduction following receptor-mediated events through the activation of transcription. STAT3 is distinctive among the STAT family of proteins in the embryonic lethality of its genetic loss, the diverse spectrum of signaling molecules that activate it, and the variability in transcription networks it activates from one cell type to another [16–20]. In addition, STAT3 is also known to have important nontranscriptional regulatory functions [21–23].

Studies utilizing strategies to directly modulate STAT3 have demonstrated that STAT3 activation protects the proximal tubular cell by limiting apoptosis in cell culture models of ischemic AKI [24] and in animal models of both nephrotoxic injury [25] and ischemic AKI [26]. Furthermore, studies have demonstrated that STAT3 signaling in kidney proximal tubule cells stimulated by the epithelial cell survival cytokine interleukin-22 (IL-22), a member of the IL-10 family of cytokines, is protective in an animal model of ischemic AKI [27, 28]. However, recent work has implicated STAT3 activation in tubular epithelial cells with the progression of kidney interstitial fibrosis [29]. Accordingly, the outcome of STAT3 activation may be dependent on timing, the context of the stimulant, and the cell in which it is activated. While the above studies begin to shed light on our understanding of STAT3 function in tubular epithelial cells during acute stress, essentially nothing is known about the function of STAT3 in kidney microvascular endothelial cells in response to stress. However, studies in cardiac IRI [30], endotoxin-induced liver injury [31], and hyperoxic lung injury [32] support the concept that endothelial STAT3 activation initiates important protective responses. Interestingly, recent work has demonstrated that JAK/STAT signaling pathways are highly upregulated in the microvascular endothelium during ischemic AKI as determined by cell-specific translational profiling [33].

In this study, we utilize a mouse with a selective genetic deletion of *Stat3* in endothelial cells to examine the effect of endothelial STAT3 signaling on injury in a model of ischemic AKI. We demonstrate that the selective loss of endothelial STAT3 signaling enhances tubular cell injury as well as the inflammatory response following IRI in the kidney. These findings suggest that targeting activation of STAT3 in the kidney microvascular endothelium during ischemic injury may serve as an important therapeutic intervention to mitigate ischemic AKI.

## 2. Materials and Methods

### 2.1. Animals and Experimental Models

All animal protocols were approved by the Indiana University Institutional Animal Care Committee and conform to the NIH Guide for the Care and Use of Laboratory Animals. Mice on a C57BL/6 background with a genetic deletion of *Stat3* restricted to the endothelium (eStat3^−/−^) were obtained as a kind gift from Dr. Xin-Yuan Fu [31]. C57BL/6 mice (WT) were obtained from Jackson Laboratory (Bar Harbor, Maine) and used as the background control. Ischemic AKI was induced using a bilateral renal artery clamp (BAC) model in C57BL/6 and eStat3^−/−^ male mice weighing 20–30 grams as previously described [34]. Sham surgery consisted of an identical procedure without placement of the microserrifine clamps to the renal arteries. For indicated experiments involving intravital imaging, *Escherichia coli* serotype 0128:B12 endotoxin (Sigma-Aldrich, St. Louis, MO) was given intraperitoneally (5 mg/kg) four hours before imaging as previously described [35]. Kidneys were harvested at the time of sacrifice and subsequently processed for further studies as described elsewhere in this section. Measurement of serum creatinine concentrations was performed at the UT Southwestern O'Brien Kidney Research Core Center.

### 2.2. Histopathology, Immunostaining, Immunoblotting, and Tissue Imaging

At the time of sacrifice, kidneys were rapidly perfusedly fixed with 4% paraformaldehyde. Tissues were subsequently processed for immunostaining or standard histochemistry. Primary antibodies to hemeoxygenase-1 (rabbit polyclonal IgG, ADI-SPA-895, Enzo Life Sciences, Farmingdale, NY), phospho-STAT3 (Tyr705, rabbit monoclonal IgG, D3A7; Cell Signaling Technology, Danvers, MA), and F4/80 (Clone CI:A3-1; Bio-Rad/AbD Serotec, Hercules, CA) were utilized for immunostaining. Appropriate secondary antibodies conjugated with Texas Red or horseradish peroxidase (HRP) were purchased from Jackson ImmunoResearch Laboratories, West Grove, PA. Negative controls were obtained by incubating kidney tissue sections from sham animals and animals undergoing renal ischemia with secondary antibodies in the absence of primary antibodies. Immunoblotting for HO-1 was performed using the above primary antibody as previously described [36]. Hematoxylin and eosin (H&E) staining of kidney tissues was performed by the Indiana University Histopathology Lab utilizing standard histochemistry procedures. Confocal immunofluorescent images of kidney tissue sections were collected at ×60 magnification using a LSM-510 Zeiss confocal microscope (Peabody, MA) or Olympus FV1000 confocal microscope (Center Valley, PA). H&E-stained images and HRP-stained images of kidney tissue sections were obtained at ×10 and ×40 magnification, respectively, with a Nikon Microphot-SA equipped with a SPOT RT Slider camera (Diagnostic Instruments Inc., Melville, NY). H&E sections were scored for tubular injury as previously described [37]. Percent of total area staining positive for HO-1 in each image was determined with Metamorph software (Universal Imaging, West Chester, PA). Tubular epithelial cells with nuclei staining positive for phospho-STAT3 and nucleated interstitial cells with staining positive for F4/80 in each field were manually counted. Tissue scoring and tissue cell counts were quantified by a blinded individual. Five to seven images of the kidney were collected from each animal.

### 2.3. Multiphoton Intravital Imaging of the Kidney

Anesthetized animals were placed on the microscope stage with the exposed intact kidney placed in a coverslip-bottomed cell culture dish bathed in warmed (37°C) isotonic saline, as previously described [38, 39]. Live animal imaging was performed using an Olympus FV1000-MPE confocal/multiphoton scanner. The scanner is equipped with a Spectra Physics Mai Tai DeepSee laser and 12-bit gallium arsenide detectors. For experiments examining kidney oxidative stress, carboxy-2′,7′-dichlorodihydrofluorescein diacetate (carboxy-DCFDA; Thermo Fisher Scientific/Invitrogen, Carlsbad, CA) was administered intravenously (7 mg/kg) 20 minutes prior to intravital imaging as previously described [35]. For experiments examining leukocyte adhesion, a fluorescein isothiocyanate- (FITC-) conjugated dextran (500 kDa; TDB Consultancy AB, Uppsala, Sweden) was injected intravenously at the time of intravital imaging to define the vascular space as previously described [39], and leukocyte adhesion to the microvascular endothelium was analyzed as previously described [40]. Quantitative analysis of acquired images was performed with Metamorph software.

### 2.4. Real-Time Quantitative PCR

RNA was extracted from snap-frozen kidneys using TRIzol. Reverse transcription on approximately 8 *μ*g of RNA was performed using the High-Capacity cDNA Reverse Transcription Kit (Life Technologies). IL-22 (Mm00444241_m1) and IL-10 (Mm004369614_m1) Applied Biosystems TaqMan gene expression assays were used. Real-time quantitative PCR amplifications were performed for 40 cycles using the 7500 Real-Time PCR Systems (Life Technologies). The ΔΔCt method was used to analyze the relative changes in gene expression. Endogenous glyceraldehyde-3-phosphate dehydrogenase (GAPDH) expression was used as a control to normalize data.

### 2.5. Statistical Analysis

Data were analyzed for statistical significance using ANOVA and pairwise *t*-tests or a nested ANOVA for nested data within the study animal. Data are reported as means with SD. Significance was set at *P* < 0.05.

## 3. Results and Discussion

### 3.1. Genetic Deletion of Endothelial Cell Stat3 Exacerbates Ischemic AKI

**Figure 1:**
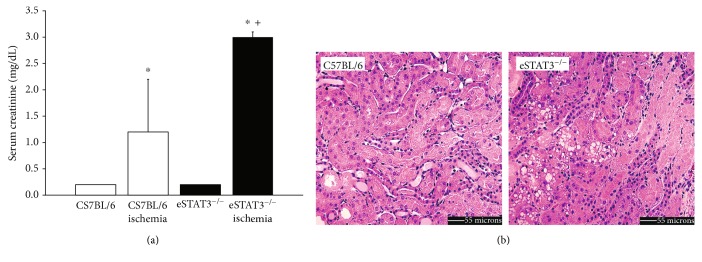
Serum creatinine 24 hours after BAC or sham surgery is depicted in (a). *P* < 0.05 compared to sham (∗) or control ischemia (+). Representative H&E images of the outer medulla 24 hours after BAC are shown in (b). Note the increased cast formation and necrosis in the eStat3^−/−^ mice.

Following induction of ischemic AKI by bilateral renal artery clamp (BAC), eStat3^−/−^ mice demonstrated a significantly greater increase in serum creatinine. Mean serum creatinine 24 hours after a 19-minute BAC was 3.0 + 0.2 mg/dL in the eStat3^−/−^ mice as compared to 1.2 + 0.9 mg/dL in the background C57BL/6 mice (Figure 1(a)). Histologic damage was also significantly greater in the eStat3^−/−^ mice as compared to the background C57BL/6 mice with mean tubular damage scores of 3.7 + 5 in the eStat3^−/−^ mice and 2.3 + 0.9 in the background C57BL/6 mice (Figure 1(b), *P* < 0.05) as defined by Kelly and coworkers [37].

### 3.2. Proximal Tubule Oxidative Stress Is Increased in eStat3^−/−^ Mice during AKI

**Figure 2:**
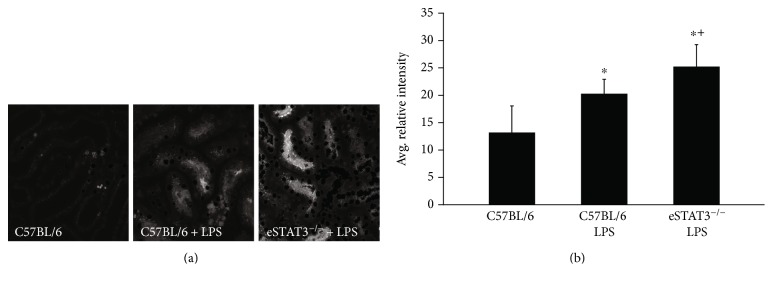
*eStat3^−/−^* and control mice were administered LPS (5 mg/kg i.p.) 4 hours prior to imaging and carboxy-DCFDA 20 minutes prior to imaging to assess tubular oxidative stress. (a) Representative images of carboxy-DCFDA fluorescence under the conditions as labeled. (b) The quantitative mean fluorescence for each condition (*n* = 3). Oxidative stress is significantly in greater degree in the *eStat3^−/−^* mice following LPS administration as compared to control mice receiving LPS (^∗^^+^*P* < 0.05) or vehicle control (^∗^*P* < 0.05).

Oxidative stress is a prominent pathophysiologic mechanism contributing to organ dysfunction in all types of acute kidney injury including ischemic AKI [41]. To ascertain the effect of the genetic deletion of *Stat3* in endothelial cells on kidney oxidative stress during injury, we utilized intravital multiphoton microscopy and carboxy-DCFDA, which is an intracellular-retained fluorescent indicator of reactive oxygen species. This can be used to visualize oxidative stress in the most vulnerable segment of the tubule, the proximal tubule [41], of the mouse kidney following injection of lipopolysaccharide (LPS) as previously described [35]. We chose to use an LPS model of AKI over the BAC model of ischemic AKI for this investigation because it provided a more consistent timing of oxidative stress for intravital imaging.  Figure 2 demonstrates that oxidative stress is significantly higher in the proximal tubules of eStat3^−/−^ mice as compared to C57BL/6 mice following LPS injection thus providing evidence that endothelial STAT3 modulates oxidative stress in the kidney, specifically the proximal tubule, during AKI.

### 3.3. Endothelial Cell Stat3 Deletion Increases Leukocyte Endothelial Cell Interactions in the Kidney during AKI and Increases Macrophage Accumulation in the Kidney during AKI

**Figure 3:**
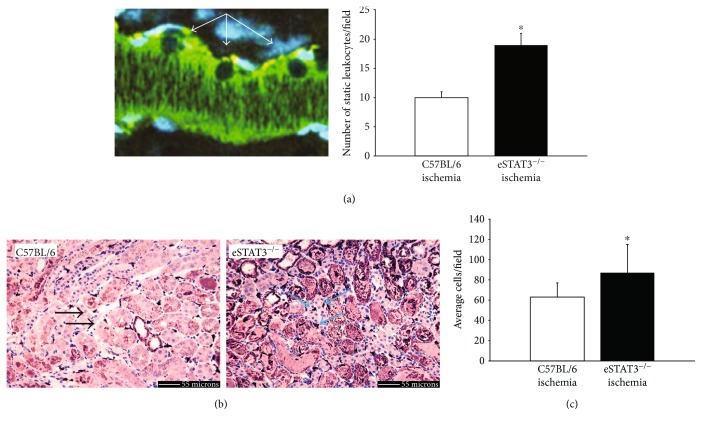
Representative intravital image of adherent or rolling leukocytes (left; courtesy of Ruben Sandoval and Dr. Bruce A. Molitoris) and quantification of adherent or rolling cells observed by intravital microscopy (right) following ischemic injury are shown in (a). Representative images of F4/80 staining following ischemic injury are shown in (b). Arrows indicate F4/80-positive cells in the interstitium representing macrophages. Average macrophage count per field is shown in (c). ^∗^*P* < 0.05.

Inflammatory responses play an important role in injury, including the promotion of oxidative stress, during ischemic AKI. Furthermore, the microvascular endothelium serves as an important coordinator of inflammatory responses during AKI [37, 42, 43] as the endothelial cell can secrete a variety of cytokines and chemokines that can activate, alert, and aim circulating inflammatory cells to injury during IRI [44–48]. Several in vivo studies have demonstrated the importance of endothelial STAT3 signaling in regulating inflammatory responses. Exaggerated proinflammatory cytokine production has been demonstrated in mouse models of cardiac IRI [30], endotoxin challenge [31], and alcoholic liver injury [49] lacking endothelial STAT3. Consequently, we next examined the impact of the endothelial *Stat3* deletion on microvascular leukocyte trafficking and tissue leukocyte composition in our models of AKI. Leukocyte adherence to the microvascular endothelium was measured using intravital microscopy by a method previously described [39, 40].  Figure 3(a) demonstrates the quantification of leukocytes rolling or adherent to the kidney microvascular endothelium during a three-minute observation period per field. Leukocytes, likely neutrophils, adherent to or rolling along the vascular endothelium of the kidney in eStat3^−/−^ mice were significantly increased as compared to those in control mice following ischemic injury. Histologic examination of tissue-resident leukocytes demonstrates that the accumulation F4/80^+^ macrophages 24 hours after ischemic injury is significantly greater in eStat3^−/−^ mice than in control mice (Figures 3(b) and 3(c)). No difference in macrophage accumulation between eStat3^−/−^ and control mice was observed following sham operation (data not shown). This finding is consistent with prior studies demonstrating increased accumulation of macrophages in mice lacking endothelial STAT3 during alcohol liver injury [49]. We did not see a difference in tissue neutrophils between eStat3^−/−^ and control mice in sham or ischemic AKI as assessed by leukocyte esterase staining (data not shown).

### 3.4. The Heme Oxygenase-1 (HO-1) Response in Proximal Tubules during AKI Is Reduced in eStat3^−/−^ Mice

**Figure 4:**
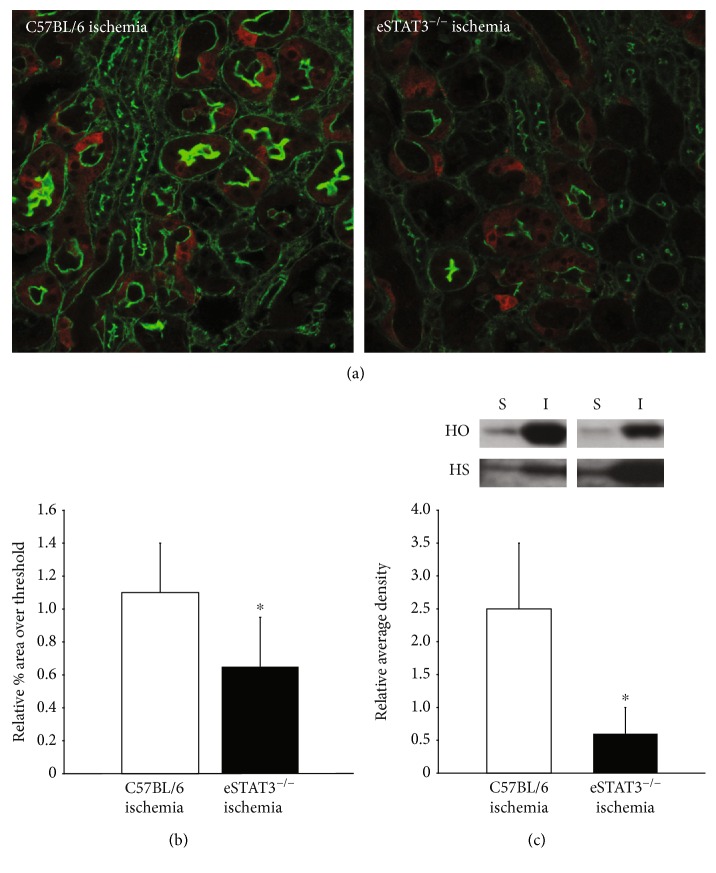
Representative images of HO-1 immunostaining (red; actin = green) in animals 24 hours after BAC are shown in (a). Immunostained images were analyzed to determine the percent area over threshold staining positive for HO-1 which is depicted in (b) relative to sham control. (c) Representative immunoblots of cortical tissue for HO-1 24 hours after BAC (HO = HO-1; HS = histone for loading control; I = ischemia; S = sham). Immunoblots were quantified by densitometry and expressed as average density normalized to histone staining and relative to sham control. ^∗^*P* < 0.05.

Heme oxygenase-1 (HO-1) is a key mediator of protective adaptations in the proximal tubule exposed to oxidative stress [50], and the beneficial effects are thought to arise from the degradation of prooxidant heme pigments released during injury, the production of antioxidant bile pigments, and the generation of carbon monoxide which has both salutary vasoactive and anti-inflammatory properties. Because expression of HO-1 is a key adaptive response for a variety of acute insults to the proximal tubule, we sought to examine the effect of the genetic deletion of *Stat3* in endothelial cells on the expression of HO-1 in the proximal tubule as a possible mechanistic link between enhanced proximal tubular injury/oxidative stress and the genetic deletion of *Stat3* in endothelial cells in AKI.  Figure 4 demonstrates that proximal tubular HO-1 expression is diminished in the eStat3^−/−^ mice as compared to the C57BL/6 mice following ischemic injury. These data suggest that endothelial STAT3 signaling is connected to the downstream generation of adaptive proximal tubular responses following injury.

### 3.5. The IL-22 mRNA Response in the Kidney Is Reduced and IL-22 Signaling in the Proximal Tubule Is Diminished in eStat3^−/−^ Mice during Ischemic AKI

**Figure 5:**
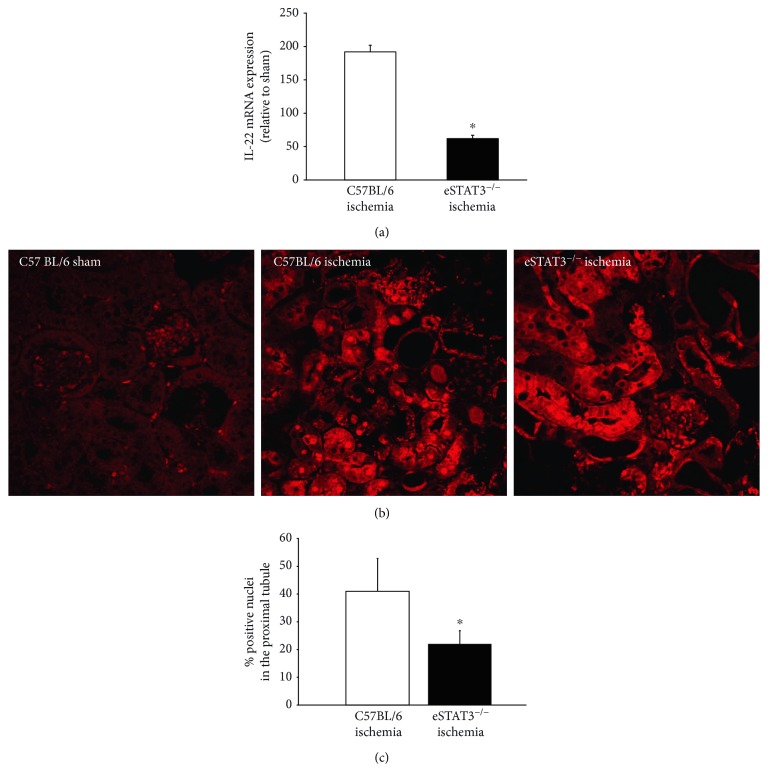
Kidney tissues harvested from *eStat3^−/−^* and control mice 24 hours after ischemic injury were processed to examine IL-22 by RT-PCR (a) and phospho (Y705)-tyrosine STAT3 (pSTAT) immunostaining as a measure of proximal tubule STAT3 activation (b, c). IL-22 gene expression (a) was normalized to GAP6DH expression and then normalized to expression in sham control. Representative images of proximal tubule pSTAT immunostaining are shown in (b). The total number of pSTAT-positive nuclei in proximal tubular cells was counted and expressed a percent of total proximal tubular cell nuclei per field (graph). ^∗^*P* < 0.05.

IL-22 is an epithelial cell survival cytokine that belongs to the IL-10 family of cytokines. IL-22 initiates signaling by binding to the heterodimer IL-10R2/IL-22R1 receptor. IL-10R2 is ubiquitously expressed whereas IL-22R1 is primarily expressed in epithelial cells of the kidney (including proximal tubule), liver, gastrointestinal tract, respiratory tract, and skin; however, it is notably absent from cells of hematopoietic origin [51]. As mentioned previously, IL-22 has been shown to be an important protective cytokine in ischemic AKI [28]. In addition, IL-22 induction of HO-1 through activation of STAT3 signaling in the proximal tubular epithelial cell is partially responsible for this protective response [52]. Furthermore, a subset of tissue-resident mononuclear phagocytes, including a subset of F4/80^+^ cells, has been demonstrated to be an important source of IL-22 during ischemic AKI [27]. Consequently, IL-22 may serve as an important mediator linking endothelial STAT3 regulation of the inflammatory response to proximal tubule injury in ischemic AKI.  Figure 5(a) demonstrates that kidney IL-22 mRNA expression in AKI is blunted in *eStat3^−/−^* mice as compared to background control (C57BL/6) mice. Figures 5(b) and 5(c) demonstrate that activation of proximal tubular STAT3 signaling, as measured by nuclear phosphor (Y705)-tyrosine STAT3 (pSTAT), is significantly reduced in *eStat3^−/−^* mice as compared to C57BL/6 mice. These data further suggest that in ischemic AKI, endothelial STAT3 regulates resident leukocyte trafficking and tissue-resident mononuclear phagocyte phenotype that in turn alters adaptive mechanisms in the proximal tubule through IL-22-mediated signaling.

## 4. Conclusions

Our findings demonstrate that loss of endothelial STAT3 signaling exacerbates injury in this rodent model of ischemic AKI. Therefore, endothelial STAT3 signaling has a protective role in ischemia-reperfusion injury of the kidney. This finding is consistent with that of the previous work done in various other models of organ injury [30–32]. As anticipated with this model of AKI, heightened tubular injury and interstitial inflammation were the predominant pathologic alterations observed. No histopathologic differences in glomeruli were noted in this study which would suggest that the endothelial STAT3 deletion differentially impacts peritubular capillaries more so than glomerular capillaries in this model, albeit more detailed studies of glomerular function would need to be performed to further characterize the consequences of the endothelial STAT3 deletion on glomerular function during injury. Our observations suggest that endothelial cell STAT3 signaling limits the trafficking of leukocytes that have the potential to exacerbate ischemic injury during AKI. Modulation of endothelial cytokine production, regulation of endothelial cytokine response, and alteration of endothelial adhesion molecule expression may all contribute to this observation and provide another fruitful avenue of future investigation. In addition, our observation that there is an increase in tissue F4/80^+^ macrophages but a decrease in IL-22 mRNA suggests that the infiltration is predominated by injurious M1 macrophages and not the reparative M2 macrophages; thus, further investigation of the phenotypic distribution of the infiltrating F4/80 macrophages is another potentially interesting path of future investigation.

Together, our findings in general underscore the complex nature of the interplay between tubular injury, inflammation, and endothelial alterations that comprise the pathophysiology of AKI and identify endothelial STAT3 as a potential regulator of this interplay. In particular, our findings suggest that endothelial STAT3 may coordinate both tubular epithelial cell adaptive mechanisms and inflammatory responses that could independently impact kidney injury following an ischemic insult. Furthermore, our findings suggest that activation of endothelial STAT3 may have important implications in the therapeutic approach to AKI; however, it is also evident that timing, degree of activation, and cell-specific targeting are additional hurdles that need to be clarified in future studies [29, 53–58].
